# Repetition Enhancement for Frequency-Modulated but Not Unmodulated Sounds: A Human MEG Study

**DOI:** 10.1371/journal.pone.0015548

**Published:** 2010-12-31

**Authors:** Linda V. Heinemann, Benjamin Rahm, Jochen Kaiser, Bernhard H. Gaese, Christian F. Altmann

**Affiliations:** 1 Institute of Medical Psychology, Goethe University, Frankfurt am Main, Germany; 2 Medical Psychology and Sociology, University Medical Center of the Gutenberg University, Mainz, Germany; 3 Institute of Cell Biology and Neuroscience, Goethe University, Frankfurt am Main, Germany; 4 International Young Scientists Career Development Organization, Graduate School of Medicine, Kyoto University, Kyoto, Japan; 5 Human Brain Research Center, Graduate School of Medicine, Kyoto University, Kyoto, Japan; Tel Aviv University, Israel

## Abstract

**Background:**

Decoding of frequency-modulated (FM) sounds is essential for phoneme identification. This study investigates selectivity to FM direction in the human auditory system.

**Methodology/Principal Findings:**

Magnetoencephalography was recorded in 10 adults during a two-tone adaptation paradigm with a 200-ms interstimulus-interval. Stimuli were pairs of either same or different frequency modulation direction. To control that FM repetition effects cannot be accounted for by their on- and offset properties, we additionally assessed responses to pairs of unmodulated tones with either same or different frequency composition. For the FM sweeps, N1m event-related magnetic field components were found at 103 and 130 ms after onset of the first (S1) and second stimulus (S2), respectively. This was followed by a sustained component starting at about 200 ms after S2. The sustained response was significantly stronger for stimulation with the same compared to different FM direction. This effect was not observed for the non-modulated control stimuli.

**Conclusions/Significance:**

Low-level processing of FM sounds was characterized by repetition enhancement to stimulus pairs with same versus different FM directions. This effect was FM-specific; it did not occur for unmodulated tones. The present findings may reflect specific interactions between frequency separation and temporal distance in the processing of consecutive FM sweeps.

## Introduction

To identify complex acoustic stimuli such as speech sounds, the auditory system has to process different components of the sound pattern in a fast and precise way. Recent magnetoencephalography (MEG), functional magnetic resonance imaging (fMRI) and psychophysical studies have investigated the processing of complex sounds such as animal vocalizations or human speech sounds [1], [2], [3]. These vocalizations vary in a number of properties, for example in terms of amplitude, frequency, and modulation rate [4]. Among these features, fast frequency modulations play a crucial role both in human speech and in animal vocalizations [5], [6]. In human speech, successful decoding of frequency variations and FM sweeps is essential for phoneme identification [7], [8], [9]. Conversely, early deficits of FM processing have been proposed to affect reading skills [10] and reduced neuronal responses to FM stimuli in a mismatch negativity paradigm in adults have been found to correlate with reading abilities [11]. A major source for understanding the cerebral implementation of FM processing are electrophysiological studies in monkeys, cats and bats. Neuronal responses to FM sweeps in primary auditory cortex have been classified according to direction selectivity and modulation rate. While most neurons respond to a broad range of modulation rates and to both upward and downward FM sweeps [12], [13], selectivity for the direction of FM sweeps could be found along the tonotopic gradient in the monkey auditory cortex. Low-frequency neurons appeared to prefer upward and high-frequency neurons downward FM sweeps [13], [14].

Earlier human psychophysical studies have shown adaptation to FM direction after repeated exposure to short FM sweeps [15] suggesting dedicated channels for FM direction coding. As these adaptation effects should be observable also at the level of mass neuronal signals, we applied a two-tone adaptation paradigm to examine neuronal computation processes in the human auditory cortex. This paradigm is based upon the neurophysiological finding that stimulus repetition reduces neural activity for several seconds. This method allows identifying the stimulus selectivity and the time course of adaptation effects in certain cortical areas. Adaptation experiments have been applied both in the visual [16] and auditory system [3], [17], [18], [19]. Besides neuronal adaptation, however, neuronal enhancement has also been observed especially in the auditory system. Two-tone experiments have revealed distinct parameter combinations leading to an enhanced neuronal response to the succeeding stimulus [17], [20], [21].

The present study assessed whether FM direction selectivity in the human cortex can be described with MEG repetition effects. We conducted an experiment which consisted of two parts: in experiment 1 pairs of frequency-modulated sweeps were presented, while unmodulated tone pairs were used in experiment 2. FM pairs consisted of sweeps with either the same or the opposite direction of frequency modulation. In either case, stimuli had the same frequency composition. We hypothesized that selectivity for FM sweep direction in the auditory cortex would lead to differential responses for stimuli with different FM directions compared to same FM directions. This difference was expected to emerge across the N1m and the P2m components as former studies had found repetition effects in both of these components e.g. [3], [22]. In experiment 2, pairs of identical vs. different unmodulated tones were used in order to examine the alternative explanation of whether repetition effects observed for the FM sweeps may be accounted for by their on- and offset parameters instead of being sweep-related.

Surprisingly, we did not observe a reduction of the neuromagnetic signal for same FM directions. In contrast, a repetition enhancement effect was observed for the sustained response starting at about 200 ms after S2. This response was significantly stronger for stimulation with the same compared to different FM direction.

## Results

### Experiment 1: FM sweeps

**Figure 1:**
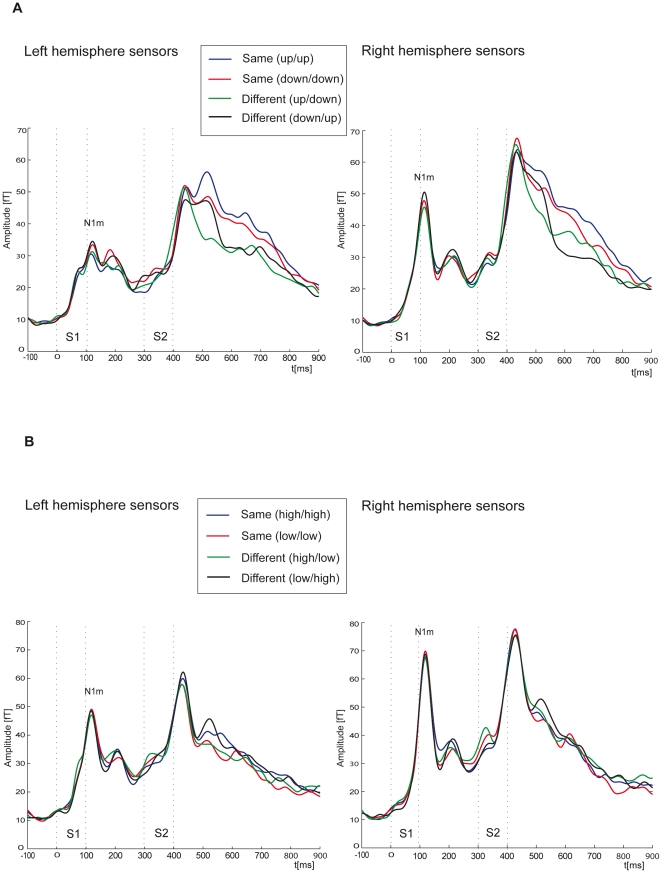
Evoked magnetic responses plotted as global field power (GFP). (A) GFP evoked by the frequency-modulated sweeps averaged across the left- and right-hemisphere sensors. (B) GFP evoked by unmodulated tones over the left- and the right-hemisphere sensors.

In experiment 1, we tested repetition effects for stimulus pairs with same or different FM direction. To quantify the MEG responses to the frequency-modulated sweeps, we calculated the GFP across all subjects for left- and right-hemisphere sensors. As shown in Figure 1A, two MEG signal components followed stimulation with S1 and S2. S1 elicited an N1m component peaking at about 113 ms after stimulus onset. For S2 the N1m component was followed by a sustained response which returned to baseline about 500 ms after S2 onset (see Figure 1A)). Mean peak amplitudes of the N1m across left-hemisphere sensors amounted to about 39 fT (sd: 8.5 fT) (mean peak latency: 102 ms, sd: 17 ms) in response to S1, whereas over the right hemisphere peak amplitudes to S1 reached 53 fT (sd: 22.7 fT) (mean peak latency: 105 ms, sd: 16 ms). Across left-hemisphere sensors N1m peak amplitudes in response to S2 amounted to about 54 fT (sd: 24.7 fT) (mean peak latency: 132 ms, sd: 17 ms), whereas across the right-hemisphere sensors they reached 68 fT (sd: 29 fT) (mean peak latency: 130 ms, sd: 15 ms). As we were interested in repetition effects, we analysed the N1m in response to the second stimulus. N1m peak amplitudes were calculated for each subject using a time window of 50 ms (100–150 ms after S2 onset). Employing a repeated measurement analysis of variance (ANOVA) with the factor hemisphere (right/left) and repetition (same/different), no differences across hemispheres (F1,9 = 4.02, P = 0.08) and for repetition (F1,9 = 0.35, P = 0.52) or interactions were observed. Mean N1m peak latencies in response to the second tone presentation (same vs. different) also did not differ between hemispheres or conditions. To compare the peak latencies in response to S1 and S2 we applied an ANOVA with the factors hemisphere (right/left), position (S1/S2) and repetition (same/different). Mean peak latencies in response to the second stimulus were significantly longer. We found a significant main effect for position (F1,9 = 31.91, P = 0.00) but no effects for hemisphere (F1,9 = 1.81, P = 0.21) or repetition (F1,9 = 1.05, P = 0.33).

**Figure 2:**
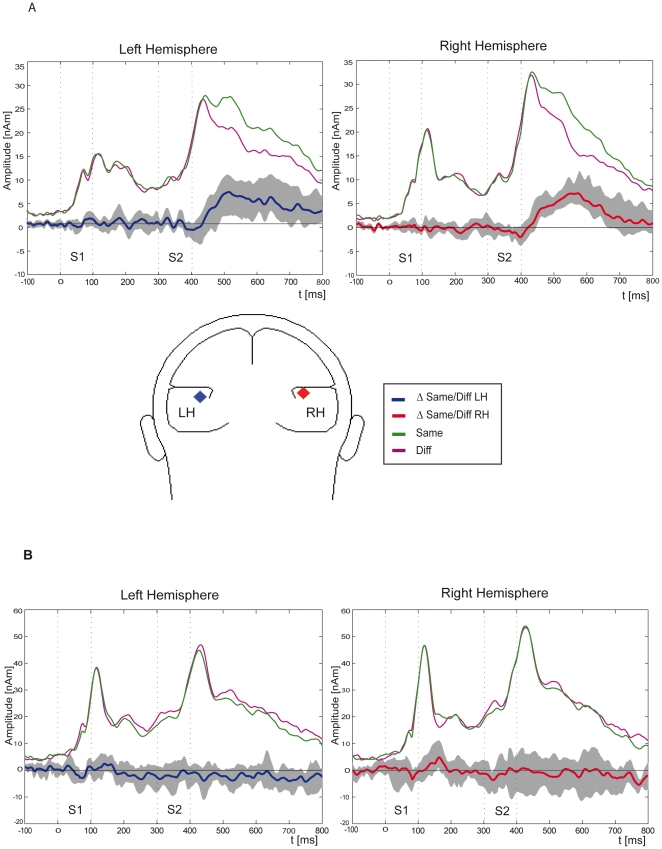
Grand average regional source waveforms. The regional sources in response to FM sweeps of same or different modulation direction have mean Talairach coordinates of (x, y, z) = −42, −16, 13 mm (sd: 7, 11, 6) (upper graphs). An example of the position for the regional sources calculated for the FM sweeps for one subject is illustrated in the center of figure 2. Grand average regional source waveforms in response to same and different unmodulated tones (mean Talairach coordinates of the sources: (x, y, z) = −36. −16, 10 (sd: 11, 13, 7)) are shown in the lower graphs. Difference waveforms are shown in blue and red for the left and right-hemisphere, respectively. The confidence range, obtained with the bootstrapping procedure, is plotted in grey. Significant differences are found mainly across right-hemisphere sensors in response to the second FM sweep at 150–350 ms after S2 onset (p<0.001, uncorrected for multiple comparisons). There were no significant differences in response to the unmodulated tones. Dotted grey lines in each of the four graphs indicate the beginning and ending of the stimuli.

To investigate repetition effects at the source level, two regional symmetric sources were fitted to each subjects' average evoked magnetic field (average across all conditions) across a time interval of 100–150 ms after S2 onset. This source model was used to explain the magnetic signal in each condition and to calculate source waveforms for each individual subject and each condition. Averaged Talairach coordinates were: (x, y, z) = −42, −17, 13 mm (sd: 7, 11, 6). As shown in Figure 2, ‘same’ and ‘different’ conditions were combined and compared by applying a bootstrapping procedure. Based on this bootstrapping statistics significant differences across right-hemisphere sensors (p<0.001, uncorrected for multiple comparisons) appeared at about 150 ms and lasted until 350 ms after S2 onset. Different results were found for left-hemisphere sensors where significant differences (p<0.001, uncorrected for multiple comparisons) only appeared at 200–300 ms after the onset of S2.These analyses revealed repetition enhancement for same compared to different FM sweeps. This effect was more pronounced in the right-hemisphere. This may suggest that the right-hemisphere plays a special role in this process.

As no differences between ‘same’ and ‘different’ conditions were observed for the N1m component but were evident for the later sustained response at 200–300 ms after the second stimulus presentation, we fitted two symmetric sources to this sustained activity. The averaged Talairach coordinates were: (x, y, z) = −41, −15, 14 mm (sd: 9, 11, 5). These two models do not differ significantly (F1,9 = 0.81, P = 0.39). Thus, the N1m source model would also show some validity for the later sustained activity.

### Experiment 2: Unmodulated tones

To control whether repetition effects for same versus different FM directions could be accounted for by the on- and offset parameters of the employed stimuli, we conducted experiment 2 with a similar design as experiment 1, but using unmodulated tones. As shown in Figure 1B, GFP of the evoked magnetic fields elicited by S1 presentation showed an N1m component starting at about 77 ms after S1 onset. S2 was also followed by an N1m component which returned to baseline at about 200 ms after S2 onset. Peak amplitudes of the N1m in response to the first unmodulated tones across left and right-hemisphere sensors amounted to about 55.7 fT (sd: 15.5) with a mean peak latency of 112 ms, sd: 15 ms and 74.8 fT (sd: 38.1) (mean peak latency: 114 ms (sd: 15 ms)), respectively. N1m peak amplitudes in response to S2 amounted to about 62 fT (sd: 22) with a mean peak latency of 124 ms (sd: 20 ms) and 80 fT (sd: 22.6) (mean peak latency: 122 ms, sd: 16 ms) for left- and right-hemisphere sensors, respectively. A repeated measurement ANOVA was conducted for mean peak amplitudes with the factors hemisphere (right/left) and position (S1/S2) and repetition (same/different). No significant differences between left and right-hemisphere (F1,9 = 4.73, P = 0.06), or in response to the first or the second stimulus (F1,9 = 1.58, P = 0.24) and for repetition (F1,9 = 0.24, P = 0.63) could be found. Analysing peak latencies, we found significantly longer latencies in response to the second tone (F1,9 = 7.39, P = 0.02), but no significant difference between right and left-hemisphere (F1,9 = 1.02, P = 0.76) and no repetition effect (F1,9 = 0.00, P = 0.94).

To model the evoked magnetic signals of the unmodulated tones, all conditions were averaged and two symmetric regional sources were fitted. For each subject this model (mean approximated Talairach coordinates: (x, y, z) = −36 -16, 10 (sd: 11, 13, 7)) was used to compare the repetition effect for unmodulated tones with the repetition of FM sweeps. ‘Same’ and ‘different’ conditions in experiment 2 were combined and the difference waveform was calculated. Figure 2 reveals that in contrast to the difference waveforms computed for the FM sweeps in experiment 1, no significant differences were observed after S2 onset. The analysis of the unmodulated tones showed neither enhancement nor inhibition effects in response to S2. This was true for both hemispheres. Similar to experiment 1, the source waveforms showed significant differences between the right and the left-hemisphere suggesting a stronger involvement of the right-hemisphere in the present type of auditory processing.

**Figure 3:**
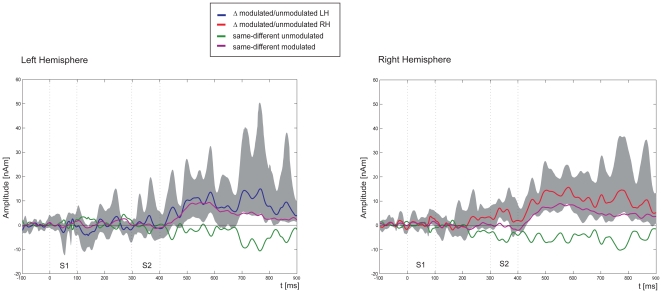
Source difference waveforms between modulated and unmodulated tones. Difference waveforms from the source models for the differences between same-different modulated and same-different unmodulated tones are shown in blue and red for the left and right-hemisphere, respectively. The confidence range, obtained with the bootstrapping procedure, is plotted in grey. Significant differences are found across right- and left-hemisphere sensors in response to the second stimulus at 150–300 ms after S2 onset (p<0.001, uncorrected for multiple comparisons). In addition, the same-different waveforms for modulated and unmodulated tones are depicted in purple and green, respectively. Dotted grey lines in each of the four graphs indicate the beginning and ending of the stimuli.

To compare repetition effects for the modulated tones with those for the unmodulated tones we calculated the difference waveforms between modulated and unmodulated conditions for each subject (see Figure 3). These difference waveforms were compared using a bootstrapping procedure. For left-hemisphere sensors significant differences (p<0.001, uncorrected for multiple comparisons) appeared at 200–300 ms and 400–500 ms after the onset of S2. Across right-hemisphere sensors significant differences (p<0.001, uncorrected for multiple comparisons) between modulated and unmodulated tones appeared at about 40–80 ms and at 130–550 ms after S2 onset.

## Discussion

This study used a two-tone paradigm to investigate selectivity for FM sweep direction in the human auditory cortex. Repetitive stimulation of selective neural populations has been proposed to result in suppression effects due to their refractoriness [23], [24]. In consideration of previous reports that have described FM-direction-selective neurons in auditory belt cortex in non-human primates [25], we had expected MEG response decrements after repeated stimulation with the same FM tone. Surprisingly, we found an enhancement of the sustained magnetic field response after FM sweep repetition. These repetition enhancement effects occurred for same compared with different FM directions in experiment 1 but not for the comparison of unmodulated tones with same versus different frequency in experiment 2. The MEG signal enhancement was observed during a time interval between 200–300 ms after S2 onset over the left hemisphere and between 150–350 ms after the second sound presentation across right-hemisphere sensors. Furthermore we observed stronger responses in right-hemisphere components about 40–80 ms after S2 onset for difference waveforms of modulated tones compared to unmodulated tones.

In the auditory system both neuronal response decrements and enhancements in response to repeated stimuli have been reported. In particular, response enhancement has been found in the macaque auditory cortex when a tone was preceded by another tone at a short interval of 70–300 ms [17], [26]. Furthermore, ERP response enhancements have been shown in humans in response to sequences of 1000-Hz tone pairs at random stimulus-onset asynchronies (SOA) between 100 and 1000 ms [27]. The strongest enhancements of the N1 peak were found for the shorter SOAs of 100–300 ms. However, several studies have found response decrements to repeated tones in terms of psychophysical detection thresholds [28], the N1 event-related potential [29] and at a later stage for the P2m component acquired with MEG [3].

Trying to determine the principles or even mechanisms underlying response enhancement to repeated presentation of identical FM stimuli (i.e with the same modulation direction) is difficult as even the representation of single FM tones has only been studied in very few investigations using electrophysiological or MEG techniques [30], [31]. This makes it hard to provide an substantial explanation that rests on previous empirical findings and goes beyond speculation.

Single-neuron studies have located the strongest interactions between consecutive tones to the cortical level [32] and a number of behavior-lesion studies in animals have suggested that tone-sequence analysis critically relies on the auditory cortex e.g. [33]. While repetitive stimulation mostly leads to response attenuation [34], several cases of response facilitation have been reported as well, mainly in long sequences with randomly varying inter-stimulus intervals (ISIs). Such facilitation was found for the N1m component and seemed to depend to a great extent on the repetition rate [35]. The ISI used in the present study (300 ms between stimulus onsets) is at the long end of the range where facilitatory interactions between auditory stimuli were found in these studies [36].

We suggest that the MEG signal enhancement effect for repetitions of identical FM sweeps may result from the interaction of two parameters, frequency separation and temporal distance between repetitions. In central auditory neurons, changes in activity across successive presentations of two sounds have been found to depend on the similarity of their frequencies: tones of similar frequency were attenuated while responses to tones with clearly deviating frequency were facilitated (see [32], Fig. 1). The repeated FM stimuli in this study might have mimicked the two-tone pattern necessary for facilitation in such a representation. Frequencies that were present at the end of the first stimulus were followed by maximally different frequencies at the onset of the second tone. That is, at a relatively short temporal distance, frequency separation was high, and may have resulted in enhancement effects. Also, similar frequencies were only present at a long temporal distance. In contrast, in stimulus pairs with the opposite sweep direction, the end of the first and the onset of the second tone were composed of identical frequencies, and stronger frequency separation was present only at a longer temporal distance. Thus, in non-repeated sweeps, frequency-wise responses may have primarily been attenuated. As a result, the observed pattern of relative response enhancement for repeated vs. non-repeated sweeps may have resulted.

Based on the same assumptions one might expect an enhancement for nonrepeated unmodulated tones (high/low; low/high conditions). There are however two differences to the FM sweeps that may explain why we did not observe these effects in our data. First, unmodulated tones may activate suppression along their temporal extent as, in contrast to FM sweeps, their frequency composition does not change over time. Second, even in the ‘different tone’ condition, only one of four frequencies that each complex tone was made up from was actually changed. Thus, in the complex tones used in this study, suppression due to similarity may have outweighed the potential enhancement effects of frequency separation. Therefore, an interaction of frequency separation and temporal distance may explain both our results in FM and unmodulated tones. It is an interaction model as the effects of frequency separation strongly depend on temporal relations.

While this scenario can account for our results, it remains speculative. However there is some support for it from the literature. As described recently, repetitive FM components in a steady-state tone can lead to increased MEG activity at a repetition rate around 4 Hz that is well compatible to the ISI used here [37]. In addition, the structure of spectral response areas as noted above [32] is directly related to the mechanisms underlying FM selectivity, as they were investigated in cortical neurons [38]. In summary, there is some support for frequency separation and temporal distance as necessary components leading to the specific enhancement in the same FM configuration.

In addition, we found a stronger response for the difference waveforms of the FM sweeps compared to the unmodulated tones. This difference appeared across the right hemisphere at 40–80 ms after stimulus presentation. One possible explanation for this finding could be the special role of the right hemisphere in the processing of frequency-modulated sounds. Both lesion studies in animals e.g. [39] and studies in epileptic patients [40] have demonstrated significant decreases in direction selectivity of FM tones when the right hemisphere is affected but not when the left hemisphere is lesioned. Also imaging studies have shown a stronger right-hemisphere involvement in an FM direction discrimination task [41], [42]. This specialization of the right auditory cortex could account for the stronger response to modulated than unmodulated sounds.

In summary our results suggest enhancement effects for tone pairs with similar FM direction and short ISIs (200 ms) but not for unmodulated tones. We hypothesize that this effect results from an interaction between frequency separation and temporal distance between the consecutively presented sounds. To corroborate this hypothesis, further studies are needed that systematically manipulate stimulus timing and frequency differences and test whether MEG enhancement effects are linked to behavioral facilitation.

## Materials and Methods

### Subjects

Participants were 10 healthy, right-handed adults (4 males, mean age 27). All subjects had normal hearing abilities as determined by self-report and reported no history of otological, neurological or psychiatric disease. Each subject gave written informed consent to participate in the study. The study was performed in accordance with the ethical standards laid down in the declaration of Helsinki of 1964. It was approved by the local ethics committee of the Goethe University Medical Faculty.

### Stimuli

**Figure 4:**
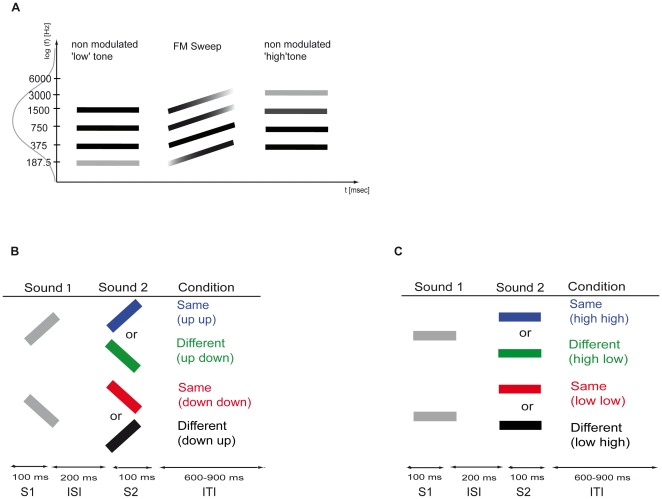
Schematic illustration of the stimuli used in both parts of the experiment and experimental conditions. (A) All stimuli consisted of four harmonic components indicated by the black bars, and were filtered with a Gaussian band-pass as symbolized by the Gaussian curve at the ordinate. The lighter gray shades of the lower and higher components indicate reduced sound intensity due to the filtering, see section on Stimuli for further details. The central sketch depicts an ascending FM sweep as used in experiment 1, the left and right sketches show the non-modulated stimuli used in experiment 2. (B) experimental conditions and procedure for the frequency-modulated sweeps, and (C) unmodulated tones.

Stimuli were created using MATLAB (The MathWorks, R2007a), with a sampling frequency of 44.1 kHz and a duration of 100 ms. All stimuli were shaped by rising and falling 5 ms ramps and were presented via insert earphones (E-A-R-tone 3A, Aearo Corporation, Indianapolis, USA) binaurally at a comfortable intensity of approximately 80–85 dB(A). In experiment 1, 20 different upward and 20 downward logarithmic frequency-modulated sweeps with a modulation rate of 10 octaves per second were used (1 octave per 100 ms). The sounds consisted of four sinusoidal components with frequencies which were separated by one octave. The different FM sweeps started at different frequencies, with the lowest rising FM sweep starting at 187.5, 375, 750 and 1500 Hz rising to 375, 750, 1500 and 3000 Hz, respectively. Each FM sweep differed in 1/20 steps of a octave from the next higher and lower FM sweep, respectively. Thus, the highest rising FM sweep started at 362, 724, 1449 and 2898 Hz and rose to 724, 1449, 2898 and 5796 Hz, respectively. We applied a logarithmic Gaussian filter with a mean of 1050 Hz and a standard deviation of 0.59 octaves (mean-sd: 698 Hz; mean+sd: 1581 Hz) for a smooth fade-out of the lowest and highest frequency complexes. These stimuli were thus a short, continuous, and glissando-like version of the Shepard's illusion [43] with the advantage that the frequency transgression from S1 to S2 was similar for sweep pairs with same and different FM directions. The experimental conditions in experiment 1 consisted of FM sweep pairs. The two sound stimuli of each pair always covered the same frequency range, that is the second stimulus of a pair was either identical to the first (“same” conditions) or a time-reversed version of the first stimulus (“different” conditions). In the “upward same” condition, an FM sweep ascending in frequency was presented twice. Similarly, in the “downward same” condition a descending FM sweep was presented twice. In the “different up/down” conditions, an ascending/descending S1 was followed by a time-reversed version of S1 (see Figure 4).

In experiment 2, 40 unmodulated sounds consisting of four sinusoidal components that contained frequencies separated by one octave. As shown in Figure 4A these frequencies were consistent with the start and end frequencies of the complex FM sounds. Two different types of stimulus pairs were presented for this experimental part: in the “same” conditions, the same complex stimulus was presented twice, either “low/low” or “high/high”. In the different conditions, S1 was followed by a complex tone S2 without the lowest but an additional higher component of S1 (low/high) or without the highest but an additional lower component (high/low). The added component was always separated by 1 octave from the lowest/highest component of S1 (see Figure 4A)).

### Procedure

The MEG experiment consisted of two parts. In experiment 1 (three runs) we investigated the processing of FM tones, while in experiment 2 (two runs) unmodulated tones were employed. Each run consisted of 361 trials and had a duration of six minutes. Subjects thus completed a randomized sequence of five runs which were separated by short breaks. Trials in both parts consisted of a two-tone paradigm following the same structure: the first stimulus (S1, 100 ms) was followed by an inter-stimulus interval (ISI, 200 ms) and the second stimulus (S2, 100 ms) (Figure 4B,4C). Trials were separated by a silent inter-trial interval of 600–900 ms. Subjects were instructed to watch a silent movie during both parts of the experiment.

Experiment 1 served to investigate frequency direction selectivity employing pairs of FM sounds of either same or different frequency modulation direction. We presented either a) two identical upward FM sweeps, b) two identical downward FM sounds (‘same’ conditions), c) an upward followed by a downward FM sound (i.e., the identical sound played in reverse order) or d) a downward followed by an upward FM sound (‘different’ conditions) (Figure 4B). All conditions were randomized across trials.

In experiment 2, pairs of unmodulated tones were presented instead of FM sweeps. The stimuli were paired according to the following conditions: a) two identical unmodulated ‘high’ tones, b) two identical unmodulated ‘low’ tones (‘same’ conditions), c) an unmodulated ‘high’ tone followed by a unmodulated ‘low’ tone and d) a unmodulated ‘low’ tone followed by a unmodulated ‘high’ tone (‘different’ conditions) (Figure 4C).

### MEG acquisition and data analysis

The neuromagnetic signals were recorded using a whole-head MEG system (CTF-MEG, VSM MedTech Inc., Coquitlam, Canada) with 275 magnetic gradiometers with an average distance between the sensors of 2.2 cm. The signals were recorded at a sampling rate of 600 Hz. Before MEG recordings, three head position indicator coils were placed at the nasion and the preauricular points and the head position was determined at the beginning and the end of each recording to ensure that head movements did not exceed 0.5 cm.

**Figure 5:**
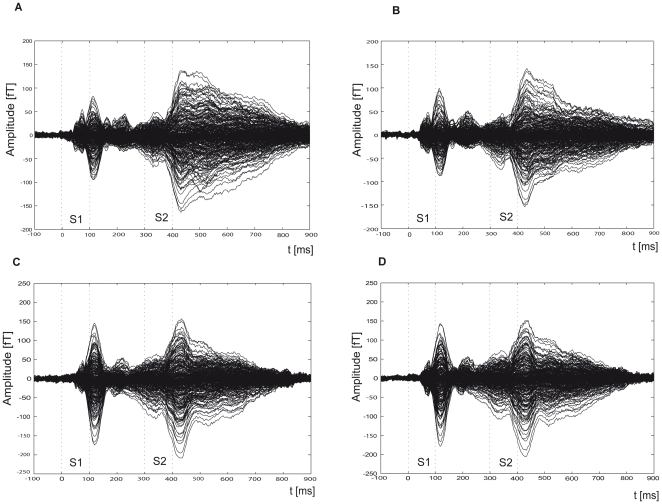
Grand-averaged event-related fields (ERF). ERFs for all subjects and sensors for the ‘same’ (on the left) and ‘different’ (on the right) conditions are shown. In parts (A) and (B) of the figure the raw data are shown for the frequency-modulated tones in experiment 1, and (C) and (D) show the ERF for the non modulated-tones in experiment 2 of the study. Dotted grey lines in each of the four graphs indicate the beginning and ending of the stimuli.

For both parts of the experiment, the MEG signals for the four conditions in experiment 1 and the two conditions in experiment 2 were averaged separately. The averaging epoch ranged from 500 ms before S1 to 1000 ms after S1 onset. A prestimulus period of 100 ms before S1 served as baseline. The data were low-pass filtered with a cutoff at 30 Hz before averaging. Epochs which contained signal variations larger than 3.5 pT were excluded from averaging. This procedure left on average 94% of the trials for further analysis. Averaged event-related fields (ERF) of all sensors for the ‘same’ and ‘different’ conditions are shown in Figure 5. For a first inspection, data were averaged across subjects and combined by calculating the global field power (GFP) [44]. The GFP was calculated by the root mean square over all right- and left-hemisphere sensors and time points for each condition. Single-subject and grand average auditory evoked potentials were computed with the BESA 5.2 software package (MEGIS software, Gräfelfing, Germany).

Source locations and time courses of source activities were computed for the average of all four ‘same’ and ‘different’ conditions for each subject separately. In experiment 1 two symmetric regional sources located in the superior temporal lobes were used to model the evoked magnetic field. The two regional sources were calculated for the N1m of the second stimulus across a time range of 100–150 ms after S2 onset. These sources were used to model the evoked magnetic fields in each condition. To test the quality of this model, goodness-of-fit (GOF) values were calculated for each condition and subject separately. Mean GOF values amounted to 85,8% (sd: 7.3) for the ‘up/up’ condition, 78.7% (sd: 5.3) for the ‘down/down’ condition, 79.7% (sd: 5.6) for the ‘up/down’ and 82.6% (sd: 8.8) for the ‘down/up’ condition. A similar data analysis was performed for the unmodulated tones used in experiment 2. Two symmetric regional sources were calculated for the averaged conditions for the N1m of S2 (time range 100–150 ms). These sources modeled 81.1% (sd: 5.9) of the evoked magnetic field of the ‘high/high’ condition, 76.4% (sd: 17.8) of the ‘low/low’ condition, and 77.1% (sd: 10.3) and 79.2% (sd: 14.4) of the ‘high/low’ and ‘low/high’ conditions, respectively.

To compare conditions, source waveforms were calculated for each subject and condition. For each subject the difference between the source waveforms of the ‘same’ and ‘different’ conditions was calculated. The rationale for comparing the ‘same’ and ‘different’ conditions rather than S1 and S2 was to overcome the ERF distortion due to the short ISI. S1 and S2 were temporally closely adjacent, resulting in a strong influence of S1 on S2. However, the “same” and “different” conditions share the same stimulus history up to S2 and thus a comparison should be unbiased by previous stimulation (for a discussion of event-related potential (ERP) distortions due to adjacent stimuli and possible solutions see [45]. To test for significant differences, a non-parametric bootstrapping procedure [46] based on 1000 iterations was applied.
